# Electrophysiologically and behaviourally active semiochemicals identified from bed bug refuge substrate

**DOI:** 10.1038/s41598-020-61368-6

**Published:** 2020-03-12

**Authors:** E. N. I. Weeks, J. G. Logan, M. A. Birkett, J. C. Caulfield, S. A. Gezan, S. J. Welham, V. A. Brugman, J. A. Pickett, M. M. Cameron

**Affiliations:** 1 Entomology and Nematology Department, University of Florida, 970 Natural Area Drive, Gainesville, Florida 32611 USA; 2 Department of Disease Control, London School of Hygiene and Tropical Medicine, Keppel Street, London, WC1E 7HT UK; 3 Vecotech Ltd, London School of Hygiene and Tropical Medicine, Keppel Street, London, WC1E 7HT UK; 4 Department of Biointeractions and Crop Protection, Rothamsted Research, West Common, Harpenden, Hertfordshire, AL5 2JQ UK; 5 VSN International Ltd, 2 Amberside House, Hemel Hempstead, HP2 4TP UK; 6 Stats4biol Consultancy, 31 Longcroft Lane, Welwyn Garden City, AL8 6EB UK; 7 School of Chemistry, Cardiff University, Cardiff, CF10 3AT UK

**Keywords:** Entomology, Electrophysiology, Chemical ecology

## Abstract

Bed bugs are pests of public health importance due to their relentless biting habits that can lead to allergies, secondary infections and mental health issues. When not feeding on human blood bed bugs aggregate in refuges close to human hosts. This aggregation behaviour could be exploited to lure bed bugs into traps for surveillance, treatment efficacy monitoring and mass trapping efforts, if the responsible cues are identified. The aim of this study was to identify and quantify the bed bug aggregation pheromone. Volatile chemicals were collected from bed bug-exposed papers, which are known to induce aggregation behaviour, by air entrainment. This extract was tested for behavioural and electrophysiological activity using a still-air olfactometer and electroantennography, respectively. Coupled gas chromatography-electroantennography (GC-EAG) was used to screen the extract and the GC-EAG-active chemicals, benzaldehyde, hexanal, (*E*)-2-octenal, octanal, nonanal, decanal, heptanal, (*R,S*)-1-octen-3-ol, 3-carene, β-phellandrene, (*3E,5E*)-octadien-2-one, (*E*)-2-nonenal, 2-decanone, dodecane, nonanoic acid, 2-(2-butoxyethoxy)ethyl acetate, (*E*)-2-undecanal and (*S*)-germacrene D, were identified by GC-mass spectrometry and quantified by GC. Synthetic blends, comprising 6, 16, and 18 compounds, at natural ratios, were then tested in the still-air olfactometer to determine behavioural activity. These aggregation chemicals can be manufactured into a lure that could be used to improve bed bug management.

## Introduction

The bed bug, *Cimex lectularius* (Linnaeus; Hemiptera: Cimicidae), is a pest of public health importance^1^. The bites associated with bed bugs are typically itchy and can lead to secondary infection, allergies, and mental health issues^2^. Furthermore, histamine has been found to be a component of bed bug faeces, and there are concerns about potential negative effects on dermal and bronchial health^3^. Prior to the discovery of dichlorodiphenyltrichloroethane (DDT) bed bugs were commonplace in homes^4^. With the use of DDT and other insecticides in homes bed bugs declined to the point that it was hard to locate a specimen. A resurgence became apparent in the 1990’s with reports published in the UK, USA and Australia^5–7^. This resurgence is mainly due to the changes in pest control practices and the development of resistance to insecticides^8–12^. With infestations being difficult to identify in the early stages and eliminate there is a need for a monitoring tool that could be used for surveillance, evaluation of intervention success and even mass trapping^13^. Traps and other monitoring tools are typically more effective if they are attractive to the target organism. The addition of semiochemicals, or behaviour and physiology modifying chemicals^14^, to a trap is likely to increase its sensitivity for detection of early stage infestations.

Bed bugs are known to form conspecific groups (aggregations) within their refuges, the formation of which is believed to be dependent on olfactory responses to semiochemicals. Aggregations of bed bugs are dynamic. As the population structure changes, the propensity to aggregate alters in a way that is population size and sex ratio dependent^15^. Filter papers that have been exposed to bed bugs (bed bug-exposed papers), which are impregnated with faeces, cuticular hydrocarbons and other compounds, have been demonstrated to be attractive in several previous studies^16–24^. As most studies have shown this attraction occurs across sexes and stages, it is likely that the response is due to an aggregation cue^24^. In contrast, extracts of conspecific exuviae were only attractive to male bed bugs, perhaps as a way to locate recently moulted virgin females^25^, and, therefore, likely do not contain aggregation cues. DeVries *et al*.^16^ showed that the response to bed bug-exposed paper was not lineage or species-specific but that the response increased according to the number of bed bugs on the paper, from one to five to 10 bugs. Previously, semiochemicals were isolated from bed bug-exposed paper by solvent extraction and from experimental bed bug refuges by air entrainment^21,22^. The extracts collected by both methods were found to be attractive to bed bugs in behavioural assays^21,22^. Through a process of elimination, a synthetic blend of 10 putative pheromone components was determined. Nonanal, decanal, (*E*)-2-hexenal, (*E*)-2-octenal, (*2E,4E*)*-*octadienal, benzaldehyde, (*R*)-limonene, (*S*)-limonene, 6-methyl-5-hepten-2-one and benzyl alcohol were reported to be important in bed bug aggregation behaviour^22^. However, the blend was not active in the absence of contact or over a distance of 30 cm^22^. This suggested that, whilst it was an attractive blend, some key components of the aggregation pheromone present in the solvent extract were missing from the synthetic mixture. Further research by Gries *et al*.^17^, managed to resolve the issue by defining an alternative blend containing volatile components, dimethyl disulfide, dimethyl trisulfide, (*E*)-2-hexenal, (*E*)-2-octenal and 2-hexanone, from headspace collections of bed bug-exposed paper and a less-volatile component, histamine, from methanol extracts of bed bug exuviae. The blend was effective at capturing bed bugs in semi-field and field studies^17^. However, due to the methods incorporated to select behaviourally relevant chemicals from the bed bug-exposed paper, for example eliminating the three esters from the blend as their removal from the blend as a group did not decrease attraction, it is possible that relevant compounds may have been missed.

As insect antennae have olfactory sensilla, recordings can be made from these sensory organs by electroantennography (EAG) and coupled gas chromatography-EAG (GC-EAG) can be used to screen volatile extracts for potential semiochemicals. Olson *et al*.^26^ used GC-EAG to identify electrophysiologically-active regions within the GC trace of a bed bug faecal extract and identified non-volatile nitrogenous compounds as putative arrestment semiochemicals. These researchers removed the antennal flagellum to stabilize the EAG signal. This terminal segment of the bed bug antenna was shown to contain the majority of the olfactory sensilla^27–29^, therefore, the study responses observed were likely due to gustatory and contact chemosensilla. The focus of Olson *et al*.^26^ was arrestment in the refuge, which was demonstrated to be reliant on the presence of the pedicel through gustatory and olfactory stimuli^19^. Although Olson *et al*.^26^ demonstrated contact chemoreception and arrestment due to the synthetic blend equivalent of their solvent extract of bed bug-exposed papers, they did not demonstrate olfaction and attraction due to volatile aggregation cues as the bed bugs were able to contact the source of the odour.

Therefore, while previous research has identified a number of bed bug aggregation cues from bed bug-exposed papers, it is unlikely that all compounds with behavioural roles in aggregation have been identified due to the fact that a full screening of the extract using the olfactory sensilla within the terminal segment of the bed bug antennae is yet to be completed. The aim of this study was to locate, identify and quantify the electrophysiologically-active (GC-EAG-active) compounds in a volatile extract collected by air entrainment from bed bug-exposed papers and determine if their behavioural role resulted in attraction.

## Results

### EAG responses of bed bugs to whole volatile extracts

To confirm the electrophysiological activity of the whole volatile extract prior to GC-EAG, standalone electrophysiological recordings were performed. *Cimex lectularius* showed EAG responses that were significantly different from those of the negative control, when tested with the 1 μL of the volatile extract, which is equivalent to 33.6 hours of air entrainment of a bed bug-exposed paper (BBEXPH) (*t*_5_ = 5.07, *P* = 0.004, 95% CI = 0.0396, 0.1211). The response to the positive control (ammonia) also was found to be significantly different from the negative control (*t*_5_ = 5.39, *P* = 0.003; CI = 0.2809, 0.7925).

### Behavioural assay 1: concentration response with the volatile extract from bed bug-exposed papers

**Figure 1:**
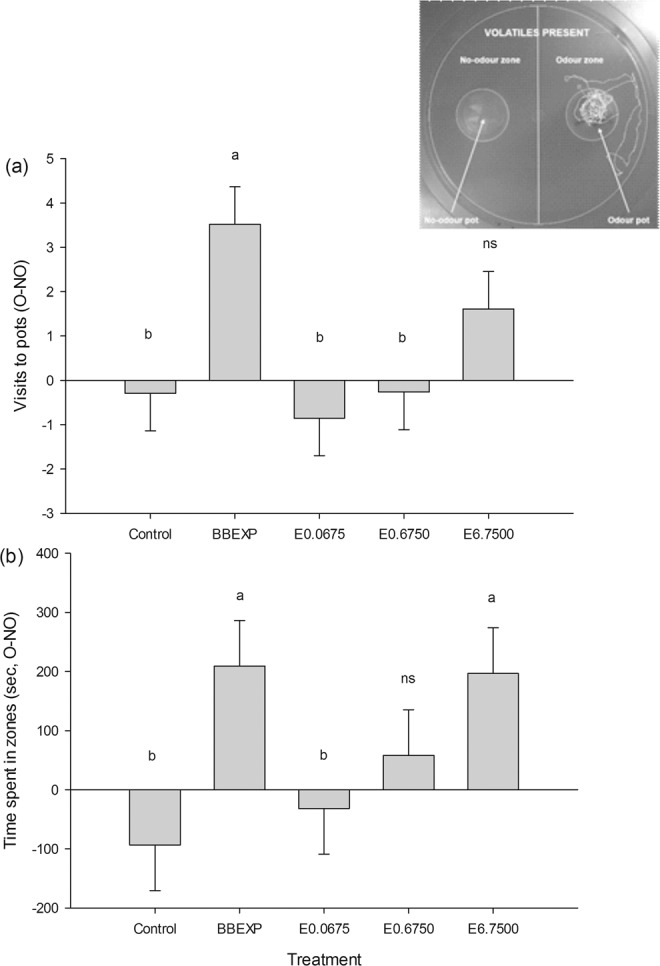
The effect of increasing concentration of the volatile extract from bed bug-exposed paper on the behaviour of *Cimex lectularius* in a still-air olfactometer (inset). Extract concentrations (E) tested were 0.0675, 0.675, and 6.75 BBEXPH (hours of bed bug-exposed paper air entrainment extract; or 0.02, 0.20 and 2.00% of the extract, respectively). The response due to a positive control of bed bug-exposed paper (BBEXP) and a negative control of solvent (re-distilled diethyl ether) are shown for comparison. Bars represent mean differences in (**a**) visits to pots (SED 1.193 visits), and (**b**) time spent in zones (s, SED 109.2 s) between odour and no-odour pots and zones, respectively. Means ± standard error; bars labelled ‘a or b’ are significantly different (at the 5% level using restricted maximum likelihood analysis) from the negative or positive control, respectively. Bars labelled ‘ns’ were not significantly different from either control.

A bioassay was conducted to test the response of male and female bed bugs to different concentrations of the volatile extract. There were significantly more visits to the odour pot (F_1,174_ = 4.73, *P* = 0.001; Fig. 1a) and significantly more time spent in the odour zone (F_1,174_ = 3.21, *P* = 0.014; Fig. 1b) in the presence of the bed bug-exposed paper (positive control) when compared with the negative control. In the presence of the volatile extract, at a concentration of 6.75 BBEXPH, bed bugs spent significantly more time in the odour zone than during controls. Only in the presence of the lowest concentration (0.0675 BBEXPH) did bed bugs spend significantly less time in the odour zone than during the positive control.

The effect of sex was not significant for time spent in zones (F_1,174_ = 3.16, *P* = 0.077). However, males of *C. lectularius* made more visits to odour pots (compared to no-odour pots) than females regardless of treatment (F_1,174_ = 4.01, *P* = 0.047). The interaction between treatment and sex was not significant for either visits to pots or time spent in zones. The proportion of bed bugs that failed to make a choice was less than 13% for all treatments.

Bed bugs spent significantly more time in the odour zone compared with the no-odour zone when the odour pot was positioned on the right-hand side of the arena (F_1,174_ = 16.21, *P* < 0.001), indicating a positional bias. However, there was no positional bias when the variable of visits to pots was analysed.

### Coupled GC-EAG responses of bed bugs to volatiles from bed bug-exposed paper

**Figure 2:**
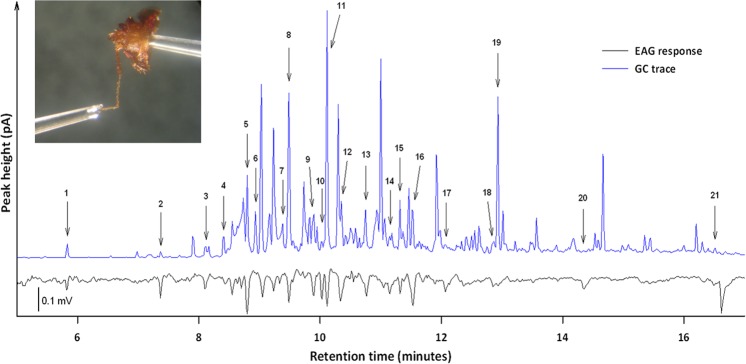
Coupled gas chromatography-electroantennography (GC-EAG) of *Cimex lectularius* (female, inset photo) responses to a bed bug-exposed paper volatile extract. Blue line corresponds to the flame ionisation detector (FID) on the GC. Black line corresponds to the antennal response of the insect preparation. Labelled peaks were GC-EAG-active in five or more of the 20 insects tested (peaks 1–21).

When the volatile extract from bed bug-exposed paper was analyzed by coupled GC-EAG, 21 GC-EAG-active peaks were recorded (peaks 1–21; Table 1, Fig. 2). Both males and females of *C. lectularius* responded to all 21 of the GC-EAG-active peaks.

**Table 1:** Identifications of electrophysiologically active peaks by coupled gas chromatography–electrophysiology (GC-EAG-active) in a volatile extract from bed bug-exposed paper.

GC-EAG-active peak	Retention Index (HP-1)	Retention Index (DB-WAX)	Chemical	Extract concentration ng/µl (n = 3)	EAG responses			Blend composition		
					Males n = 10	Females n = 10	Total (n = 20)	SB-6	SB-16	SB-18
1	n/c	5.84	Hexanal	14.61	5	4	9	•	•	•
2	880	7.38	Heptanal	4.92	6	7	13	•	•	•
3	933	8.12	Benzaldehyde	4.84	5	3	8		•	•
4	965	8.56	(*RS*)-1-Octen-3-ol	25.95	4	7	11		•	•
5	983	8.81	Octanal	73.42	9	10	19	•	•	•
6	1000	9.06	3-Carene	28.03	4	7	11		•	•
7	1023	9.34	β-Phellandrene	28.98	5	6	11		•	•
8	1036	9.50	(*E*)-2-Octenal	102.23	8	8	16	•	•	•
9	1068	9.90	(3*E,5E*)-Octadien-2-one	43.35	8	5	13			•
10	1078	10.03	Not Identified	n/a	8	9	17			
11	1085	10.12	Nonanal	266.15	8	9	17	•	•	•
12	1104	10.36	Not Identified	n/a	7	10	17			
13	1140	10.77	(*E*)-2-Nonenal	67.68	5	8	13	•	•	•
14	1173	11.15	2-Decanone	19.47	5	4	9		•	•
15	1187	11.32	Decanal	60.63	7	5	12		•	•
16	1205	11.53	Dodecane	36.17	8	8	16		•	•
17	1256	12.08	Nonanoic acid	33.07	6	6	12		•	•
18	1331	12.88	2-(2-Butoxyethoxy)ethyl acetate	63.26	7	5	12		•	•
19	1337	12.94	(*E*)-2-Undecenal	16.74	4	5	9		•	•
20	1479	14.35	(*S*)-Germacrene D	0.67	7	7	14			•
21	1723	16.52	Not Identified	n/a	4	3	7			

Peaks identified tentatively by coupled gas chromatography–mass spectrometry (GC–MS), confirmed by peak enhancement with authentic chemical standards and quantified at 100% by multiple point external standards. One microliter is equivalent to 33.6 hours of bed bug-exposed paper air entrainment (BBEXPH).

Number of responses by electroantennogram (EAG) by males and females and the composition of the three synthetic blends (SB) with 6 (SB-6), 16 (SB-16), and 18 (SB-18) GC-EAG-active chemicals tested is provided (black circles indicate chemicals present in each synthetic blend). The retention index of GC-EAG-active peak 1 was not calculable (n/c) as the alkane range did not extend this far into the trace on the HP-1 column.

### Identification and quantification of GC-EAG-active compounds

Following tentative identification by coupled gas chromatography-mass spectrometry (GC-MS), identifications were confirmed by co-injection of the authentic compounds with the extracts on HP-1 and DB-WAX columns (Table 1). Of the 18 compounds tentatively identified by coupled GC-MS, 17 were successfully confirmed by peak enhancement on both columns. Nonanoic acid (peak 17) was an exception, due to poor resolution of acids on HP-1 columns, the co-injection was only valid on the DB-WAX column. Three compounds were not successfully identified by coupled GC-MS (GC-EAG-active peaks 10, 12 and 21).

Multiple point external standards of the 18 chemicals identified by coupled GC-MS and confirmed by peak enhancement were used to produce calibration curves for quantification. The equation of the response curve was used to calculate the quantity of each chemical that was present in 1 µL (33.6 BBEXPH) of the volatile extract from bed bug-exposed papers (Table 1). The coefficient of determination or R^2^ values for the linear regressions completed for all quantifications were greater than 0.98. The concentrations of the identified chemicals within the extract ranged from 0.67 ng/µL of (*S*)-germacrene D to 266.15 ng/µL of nonanal. Nonanal was a major component of the blend along with (*E*)-2-octenal at 102.23 ng/µL.

### Behavioural assay 2: concentration response with the 16-component synthetic blend

**Figure 3:**
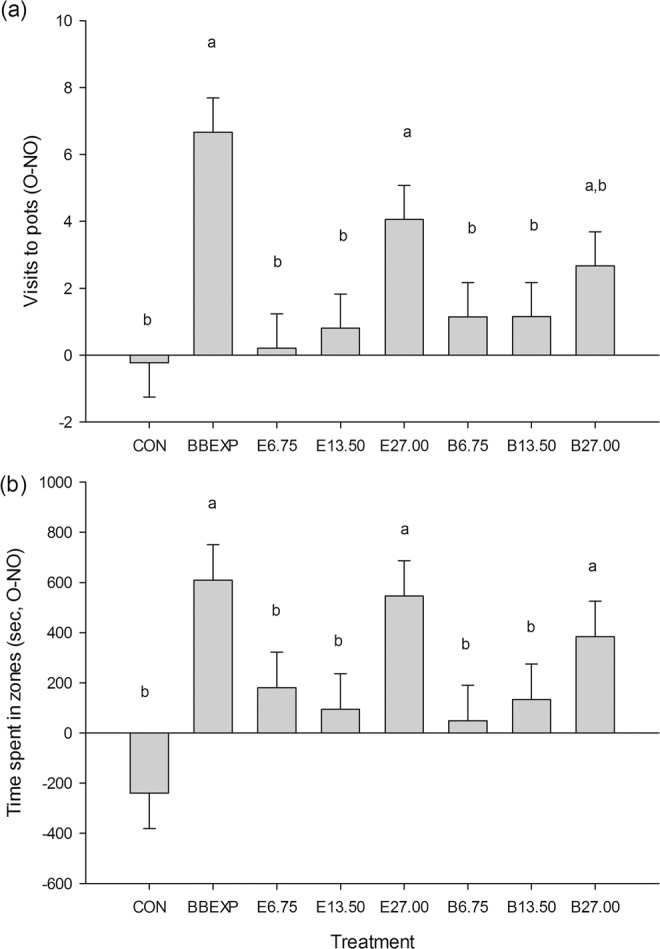
The effect of concentration of volatile extract (E) and a 16-component synthetic blend (B) on the behaviour of *Cimex lectularius* in a still-air olfactometer. Concentrations tested were 6.75, 13.50, and 27.00 BBEXPH (hours of bed bug-exposed paper air entrainment extract; or 2, 4 and 8% of the extract, respectively) for both extracts and blends. The response due to a positive control of bed bug-exposed paper (BBEXP) and a negative control of solvent (re-distilled diethyl ether, CON) are shown for comparison. Bars represent mean differences in (**a**) visits to pots (SED 1.428 visits), and (**b**) time spent in zones (s, SED 206.5 s) between odour and no-odour pots and zones, respectively. Means ± standard error; bars labelled ‘a or b’ are significantly different (at the 5% level using restricted maximum likelihood analysis) from the negative or positive control, respectively.

Here, we tested the response of male and female bed bugs to different concentrations of the synthetic blend to determine the optimum concentration for attraction to the blend when compared to the volatile extract. There were significantly more visits to the odour pot (F_7,118.5_ = 5.28, *P* < 0.001; Fig. 3a) and significantly more time spent in the odour zone (F_7,136_ = 3.72, *P* = 0.001; Fig. 3b) in the presence of the bed bug-exposed paper (positive control) when compared with the negative control. In the presence of the volatile extract or synthetic blend at a concentration of 27.00 BBEXPH, bed bugs spent significantly more time in the odour zone and made more visits to the odour pot than during negative controls. At the lower concentrations, for both the volatile extracts and the synthetic blends, there were significantly fewer visits to the odour pot and time spent in the odour zone compared with the positive control.

The effect of the position of the odour pot did not have a significant effect on either time spent in zones (F_1,136_ = 0.01, *P* = 0.915) or visits to the pots (F_1,118.5_ = 0.62, *P* = 0.434). The number of bed bugs that failed to make a choice was less than 25% for all treatments.

### Behavioural assay 3: testing different synthetic blends

**Figure 4:**
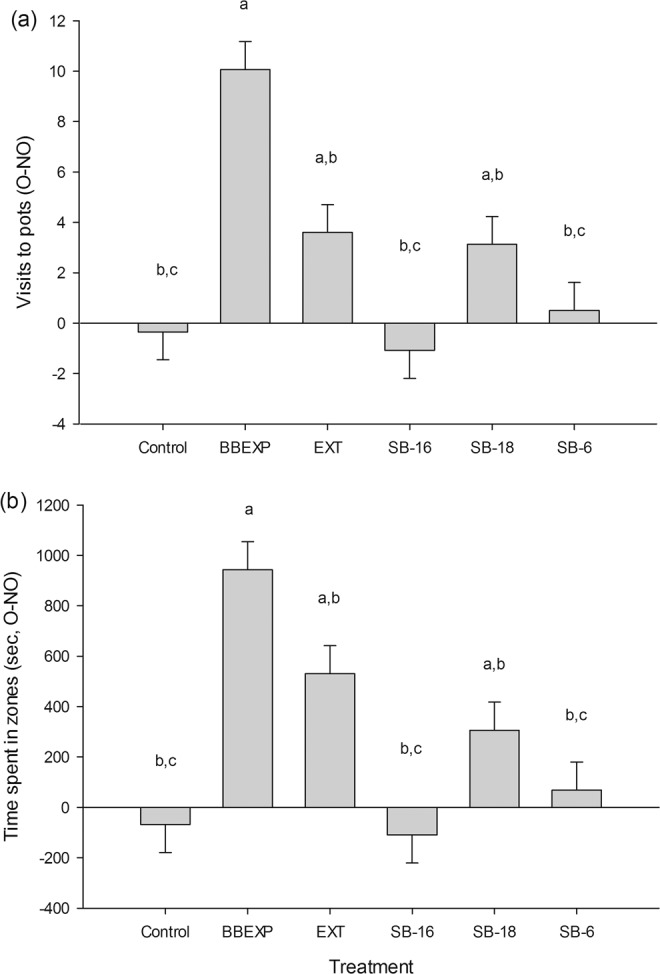
The effect of 16-, 18-, and 6-component synthetic blends (SB-16, SB-18, and SB-6, respectively) on the behaviour of *Cimex lectularius* in a still-air olfactometer. Synthetic blends (27 BBEXPH, i.e. hours of bed bug-exposed paper air entrainment extract; or 8% of the extract) tested and compared to the response due to two positive controls, bed bug-exposed paper (BBEXP) and a volatile extract (EXT) from bed bug-exposed paper (27 BBEXPH), and a negative control of solvent (re-distilled diethyl ether). Bars represent mean differences in (**a**) visits to pots (SED 1.586 visits) and (**b**) time spent in zones (s, SED 158.8 s) between odour and no-odour pots and zones, respectively. Means ± standard error; bars labelled ‘a,b or c’ are significantly different (at the 5% level using restricted maximum likelihood analysis) from the negative control, positive control or volatile extract, respectively.

In this assay, we attempted to narrow down the synthetic mixture to fewer components by testing the response of male bed bugs to a 6-component synthetic blends (SB) when compared to the 16-component synthetic blend tested in behavioural assay 2 and the full 18-component blend. There were significantly more visits to the odour pot (F_5,80.4_ = 9.20, *P* < 0.001; Fig. 4a) and significantly more time spent in the odour zone (F_5,87_ = 10.51, *P* < 0.001; Fig. 4b) in the presence of bed bug-exposed paper (positive control), the volatile extract from bed bug-exposed paper and SB-18, when compared with the negative control. The number of visits to the odour pot and the time spent in the odour zone was significantly greater in the presence of the bed bug-exposed paper than for any of the other treatments. The volatile extract was significantly more attractive than SB-16, SB-6 and the negative control. There was no significant difference in the number of visits to the odour pot and the time spent in the odour zone in the presence of the volatile extract and SB-18.

The effect of the position of the odour pot did not have a significant effect on either time spent in zones (F_1,87_ = 0.03, *P* = 0.853) or visits to the pots (F_1,86.2_ = 1.49, *P* = 0.226). The number of bed bugs that made no choice was less than 20% for all treatments. In fact, only in the presence of SB-6 were there any bed bugs that failed to make a choice, in these bioassays four of the 20 individuals tested made no choice.

## Discussion

Since the behavioural and electrophysiological characterisation of the bed bug alarm pheromone^27^, there have been only a few studies in bed bug chemical ecology that have utilised EAG and only a single study that has utilized GC-EAG^26^. In the current study, when a volatile extract, collected from bed bug-exposed papers by air entrainment, was tested by EAG, bed bugs showed a response that was significantly different from the response to controls. This result provides evidence that the air entrainment successfully collected GC-EAG-active volatiles from bed bug-exposed papers. Coupled GC-EAG was used to analyse this volatile extract with the aim of identifying the compounds responsible for the attraction and arrestment to bed bug-exposed papers that has been observed in previous behavioural studies^16–24^. Aggregation pheromones, by definition, should be detectable by males, females, and nymphs, as all stages and both sexes aggregate^30^. Although nymphs were not tested, both males and females were found to respond to the same 21 peaks by coupled GC-EAG, which indicates that the compounds cause a response that is relevant to both sexes, such as aggregation. However, further behavioural assays are necessary to determine the behavioural role of each GC-EAG-active compound.

In several laboratory-based experiments, the behavioural activity of a volatile extract collected by air entrainment from bed bug-exposed paper was investigated. At the highest concentration tested (8% or 27 BBEXPH), the volatile extract initiated orientation towards the pot that had been treated with the extract, showing attraction to the volatiles that was significantly different to the response to the negative control. This demonstrates that the isolation technique of air entrainment successfully collected the key volatiles that were responsible for the attraction to the behaviourally-active material, i.e. the bed bug-exposed paper. Air entrainment has been used previously to isolate volatiles from bed bug-exposed paper^17,22^. However, due to differences in behavioural assay and chemical isolation techniques, all three studies, including the current study, have identified a different array of potential semiochemicals, but with some chemicals in common.

The semiochemical blend identified in this study includes one aromatic aldehyde, one alcohol, one alkane, one carboxylic acid, one ester, one sesquiterpene, two ketones, two monoterpenes, and eight alkyl aldehydes. Although the majority of the chemicals identified in this study have been described before as semiochemicals of other insects with similar feeding and aggregation behaviour, only a few have been previously described with relevance to bed bug chemical ecology (benzaldehyde, hexanal, (*E*)-2-octenal, octanal, nonanal and decanal)^22,31^. The majority of the compounds identified in the present study, as potential semiochemicals for bed bugs, have been recorded here for the first time, including: heptanal, (*R,S*)-1-octen-3-ol, 3-carene, β-phellandrene, (*3E,5E*)-octadien-2-one, (*E*)-2-nonenal, 2-decanone, dodecane, nonanoic acid, 2-(2-butoxyethoxy)ethyl acetate, (*E*)-2-undecanal and (*S*)-germacrene D. Of the compounds that have been reported as semiochemicals previously, 13 compounds: hexanal, heptanal, benzaldehyde, 1-octen-3-ol, octanal, 3-carene, (*E*)-2-octenal, nonanal, (*E*)-2-nonenal, decanal, dodecane, (*E*)-2-undecenal and nonanoic acid, were identified as promoting aggregation behaviour in insects^22,32–40^. Whereas, 1-octen-3-ol, (*E*)-2-nonenal and 2-decanone, are compounds of importance for host location and blood feeding in other haematophagous insects^41–54^. Hexanal, heptanal, octanal, nonanal, (*E*)-2-octenal and decanal, have been previously identified as compounds used for insect defence^55,56^. Only two of the compounds identified in the present study, (*3E,5E*)-octadien-2-one and 2-(2-butoxyethoxy)ethyl acetate, had no previous record in insect chemical ecology. Indeed, it is appreciated that 2-(butoxyethoxy)ethyl acetate is a synthetic industrial chemical but, because it has biological activity and is not in the control extracts, it has been treated as a bed bug associated compound in this study.

Benzaldehyde, (*E*)-2-octenal, nonanal and decanal, were found in a previous study to be components of a potential airborne bed bug aggregation pheromone, along with (*E*)-2-hexenal, (*2E,4E*)-octadienal, (*R*)- and (*S*)-limonene, 6-methyl-5-hepten-2-one and benzyl alcohol^22^. The compounds were identified from volatile extracts from experimental bed bug refuges and were subsequently shown to induce significant behavioural responses in bed bug nymphs when presented as part of a 10-component synthetic blend^22^. In the same study, octanal, along with (*2E,4Z*)-octadienal, benzyl acetate and geranylacetone, was identified from the volatile extracts^22^. However, all four compounds were removed from the blend after subtraction experiments revealed them to have no significant additive effect on the behaviour of bed bug nymphs^22^. Gries *et al*.^17^ defined a blend containing (*E*)-2-octenal along with additional volatile components dimethyl disulfide, dimethyl trisulfide, (*E*)-2-hexenal, and 2-hexanone and a less-volatile component, histamine, which was effective at capturing bed bugs in semi-field and field studies. Gries *et al*.^17^ defined histamine as a non-volatile component of the bed bug aggregation chemical. However, we were unable to incorporate its testing into the current study as the experiments detailed herein were completed before its publication. In future studies it would be interesting to compare the blend defined by Gries *et al*.^17^ with the blend defined in this study, as well as testing our blend in combination with histamine. Hexanal, benzaldehyde and (*E*)-2-octenal also were identified from excreta of tropical bed bugs, *C. hemipterus*, in behavioural assays and all three compounds were attractive at certain concentrations^31^.

(*E*)-2-Octenal, along with (*E*)-2-hexenal, are the major constituents of the bed bug alarm pheromone^55^. The defence secretion, from the metathoracic scent glands also contains butanone, acetaldehyde, 4-oxo-(*E*)-2-hexenal and 4-oxo-(*E*)-2-octenal in minor amounts^55,57^ and trace amounts of nonanal, along with 2,4-octadienal^57^. Despite being the most abundant compounds in headspace collections from experimental bed bug refuges, (*E*)-2-octenal and (*E*)-2-hexenal were at much lower concentrations there than in the head space above mechanically disturbed bed bugs^22^. As well as aggregation behaviour, an aggregation pheromone may affect other physiological and behavioural factors or have multifunctionality. For example, the aggregation pheromone of the southern green stink bug, *Nizara viridula* (Linnaeus; Hemiptera: Pentatomidae), is believed to act as a defence secretion or alarm pheromone at high concentrations^58^. A concentration-response to (*E*)-2-octenal and (*E*)-2-hexenal was demonstrated in bed bugs by Ulrich *et al*.^59^; both chemicals were attractive at a low concentration but repellent at higher concentrations. Therefore, it is believed that (*E*)-2-octenal along with (*E*)-2-hexenal could be multifunctional, i.e. affecting more than one aspect of bed bug behaviour or physiology, by acting as an alarm pheromone at high concentrations but as an aggregation pheromone at lower levels^22,59^.

The compounds identified in this study are bed bug-derived and the fact that they are GC-EAG-active implies a purpose for them in bed bug behaviour. Testing of the synthetic blends of GC-EAG-active compounds revealed that a significant response in a behavioural assay can be achieved at a concentration of 8% (or 27 BBEXPH). In behavioural assay 2, SB-16 was found to be attractive and in behavioural assay 3, SB-18 resulted in significant attraction. In behavioural assay 3, neither SB-16 nor the 6-component blend (SB-6) caused significantly greater attraction than the negative control. The lack of response to SB-6 and SB-16, in behavioural assay 3, could imply that some or all of the additional compounds in SB-18, i.e. benzaldehyde, (*RS*)-1-octen-3-ol, 3-carene, β-phellandrene, 2-decanone, decanal, dodecane, nonanoic acid, 2-(2-butoxyethoxy) ethyl acetate, (*E*)-2-undecenal, (*S*)-germacrene D and (*3E,5E*)-octadien-2-one, were essential for attraction.

Attraction to the volatile extract was often less than the response to the bed bug-exposed papers. For example, in behavioural assay 3 the bed bug-exposed paper was significantly more attractive than all other treatments. The result implies that there are possibly additional components to the bed bug-exposed paper that have not been identified from the volatile extract. Although the volatile extract and synthetic blends were applied at a concentration that was equal to 20 min of volatile production by the bed bug-exposed paper, it was not possible to control for the difference in release rate. Papers that have been exposed to bed bugs remain attractive over long periods of time and tests on the same paper have shown no decrease in attraction over periods of months (personal observation). In contrast, clean unexposed filter papers that have been treated with a volatile extract from bed bug-exposed paper were not attractive when left for 20 min prior to the initiation of the bioassay (personal observation). If all the chemicals that make up the multi-component attractant are present and in the correct ratios, this variation in response could be attributed to the difference in release rate between the solvent-based volatile extract when compared with the bed bug-exposed papers themselves. As the substrate in all cases was filter paper, there may be additional non-volatile compounds on the bed bug-exposed paper that are acting as a slow-release matrix, ensuring a steady release of the semiochemicals is achieved. For effective use in the field it will be necessary to formulate the blend so that the release rate is controlled, and the chemicals are protected from environmental degradation.

In this study, it has been confirmed that the volatile extract from bed bug-exposed papers, at a concentration of 8%, that corresponds to 27.00 hours of bed bug-exposed paper entrainment time (BBEXPH), is consistently attractive to bed bugs in an olfactometer. Volatiles that the bed bug can detect through olfaction have been identified and 18 compounds have been confirmed to be volatiles with importance in bed bug chemical ecology. There is strong evidence that the GC-EAG-active chemicals identified in the present study are involved in bed bug aggregation, since they were obtained from a behaviourally-active source. Although many of the chemicals have been described before, with relevance to the chemical ecology of haematophagous insects or aggregation behaviour, only a few have been described with relevance to bed bugs. In the present study, heptanal, (*R,S*)-1-octen-3-ol, 3-carene, β-phellandrene, (*3E,5E*)-octadien-2-one, (*E*)-2-nonenal, 2-decanone, dodecane, nonanoic acid, 2-(2-butoxyethoxy)ethyl acetate, (*E*)-2-undecanal and (*S*)-germacrene D have been described for the first time as putative bed bug semiochemicals. Additionally, this is the first report in the literature of 2-(2-butoxyethoxy) ethyl acetate or (*3E,5E*)-octadien-2-one as putative insect semiochemicals. The synthetic blend containing all 18 components was attractive to bed bugs. Further experiments to refine the blend are necessary before moving on to test these putative semiochemicals in the field. Future work will concentrate on developing a suitable formulation to achieve the desired rate of release for extended periods. Although our knowledge of bed bug chemical ecology is ever increasing, there are few bed bug monitoring tools on the market that have been adopted for use by the pest control industry or public. An attractive semiochemical lure used to bait a trap would provide a valuable tool for management of bed bugs to enable surveillance for targeted control efforts and evaluation of treatment success as well as the potential for population suppression of this public health pest.

## Materials and Methods

### Insects

Rearing followed procedures outlined in Weeks *et al*.^23,24^. In brief, *C. lectularius* were obtained from a colony at the University of Sheffield (UK) and then reared at Rothamsted Research (UK). Bed bugs were reared in colony pots in a controlled environment at 25 °C ± 1.5 °C and 80% RH ± 5%. The colony was maintained in 60 × 40 mm plastic pots. A 20 mm diameter hole was made in the centre of the plastic screw top lid to permit air exchange. A piece of fine mesh was held in place between the pot and the lid with a piece of elastic, this prevented bed bugs from escaping and permitted removal of the lids to facilitate feeding. The light regime was set to 14L:10D. Once per week bed bugs were given access to heparinised sheep blood (TCS Biosciences, Botolph Claydon, UK) using glass feeders^60^. These feeders were cleaned after use with warm water with detergent (1% Teepol 12–20 unperfumed detergent, Hertfordshire supplies, Welwyn Garden City, UK) followed by an acetone rinse before being placed at 150 °C for at least 12 h.

Experimental insects, unless otherwise stated, were adults of both sexes that had been blood-fed 7 to 14 days previously. This stage of bed bugs was easier to handle without damage and were more active in the arena than recently fed bed bugs. Twelve hours before use, experimental insects were transferred into a pot and moved into the behaviour room to acclimatise. Bed bugs were chosen randomly from colony pots, identified as adults and sexed under a dissecting microscope. Both males and females of *C. lectularius* were used for behavioural assays testing volatile extracts. However, as there was no significant difference in response to the volatiles from bed bug-exposed papers between the sexes in previously published experiments^24^, only males of *C. lectularius* were used to confirm the behavioural activity of the synthetic blends. All experiments were conducted between the years of 2007 to 2011.

### Isolation of volatiles from bed bug-exposed papers

#### Bed bug-exposed papers

Bed bug-exposed papers used for volatile isolation were filter papers that had previously been exposed to bed bugs. One hundred bed bugs of mixed sex and stage were put into plastic colony pots containing filter papers (accordion folded, 70 × 40 mm, Whatman 125 mm, Whatman PLC, Maidstone, UK). Bed bugs used the paper as a refuge; aggregating, defaecating and ovipositing in the folds. After one month, the bed bugs, eggs and exuviae were removed from the paper and the paper was tested for behavioural activity using the still-air olfactometer described by Weeks *et al*.^24^. Gloves were worn whilst handling the bed bug-exposed paper.

#### Isolation of volatiles from bed bug-exposed papers

Volatiles from bed bug-exposed papers were isolated by air entrainment. Ten bed bug-exposed filter papers were contained in a glass vessel (250 mL). Ten unexposed filter papers (accordion folded, 70 × 40 mm, Whatman 125 mm) were placed in a second vessel as a filter paper control. As a blank control, an entrainment also was completed of an empty glass vessel. Air was pulled through a charcoal filter (activated charcoal, 10–14 mesh, 50 g, BDH Chemical Ltd, Poole, UK) for purification, into the glass chambers and across the filter paper at a flow rate of 600 mL/min. Air from each vessel was then pulled out into a Pyrex tube containing Porapak Q (50 mg, mesh size 50/80, Supelco, Bellefonte, PA, USA), a porous polymer that traps volatile compounds, held in place by two plugs of silanised glass wool (Supelco, Bellefonte, PA, USA). Entrainment vessels were purged for 10 min prior to insertion of the Porapak tubes and then the entrainment was run for 7 days. When the Porapak tubes were removed, they were eluted with 750 μL of freshly re-distilled diethyl ether into 1.1 mL pointed vials (Chromacol, Welwyn Garden City, UK). The vials were stored at −20 °C prior to analysis. The entrainment was repeated eight times. As each entrainment contained ten papers, each extract contained the equivalent of 1680 bed bug exposed paper hours or BBEXPH. As the extracts were 500 uL in volume they contained 3.36 BBEXPH per uL. Charcoal filters were cleaned before entrainment by attachment to a flow of purified nitrogen whilst being heated in an oven (150 °C) for two hours. Porapak tubes were cleaned by washing with dichloromethane (1 mL) followed by re-distilled diethyl ether (4 mL) and conditioned by attachment to a flow of purified nitrogen whilst in heating blocks (135 °C) for two hours. All glassware was washed in warm water with detergent (1% Teepol 12–20 unperfumed detergent), then acetone and placed in an oven (150 °C) for two hours before being used.

#### Gas chromatography

Extracts were analysed using a Hewlett Packard (HP-6890) GC with a non-polar (HP-1) and a polar (DB-WAX) column (J & W Scientific, Folsom, CA, USA). HP-1 (50 m × 0.32 mm; film thickness, 0.52 μm) and DB-WAX (30 m × 0.32 mm; film thickness, 0.5 µm) columns were fitted with a cool-on-column (COC) injector, hydrogen carrier gas and a flame ionisation detector (FID). The oven temperature was maintained at 30 °C for 0.5 min and then programmed at 5 °C/min to 150 °C, held for 0.1 min, then 10 °C/min to 230 °C and then held for 35 min.

Compounds within the samples were quantified approximately by comparison with an injection (1 μL) of 100 ng/μL solution of n-alkanes (C_7_-C_25_) in hexane, and then concentrated to 50 µL so that the largest peak was at approximately 100 ng/μL, using a gentle flow of purified nitrogen before being re-analysed. As each extract was concentrated 10-fold, from 500 uL to 50 uL, the extracts now contained 33.6 BBEXP per uL. Retention indices (RI) were calculated following Bartle^61^. An aliquot (30 μL) of each of the eight volatile extracts were combined for further analysis.

### Electrophysiology

#### Insect preparation

The electrodes used to make recordings were Ag/AgCl wires (diameter 0.37 mm; Harvard Apparatus, Edenbridge, UK) inserted into glass pipettes, made from borosilicate glass capillaries (outer diameter 2.00 mm; inner diameter 1.16 mm; Harvard Apparatus, Edenbridge, UK). The glass pipettes were filled with Ringer’s solution (7.55 g NaCl, 0.64 g KCl, 0.22 g CaCl_2_, 1.73 g MgCl_2_, 0.86 g NaHCO_3_ and 0.61 g Na_3_PO_4_ per L water). Before connecting the insect, the tips of the glass pipettes/electrodes were put into contact to confirm electrical connectivity. The bed bug was then chilled on ice for one minute before dissection. The head was removed by an incision through the pronotum allowing the thorax and abdomen to be discarded. The proboscis and one antenna were amputated to reduce noise. One of the glass pipettes was then inserted into the back of the head, through the pronotum, and the tip of the remaining antenna was inserted into the recording electrode that was already in position (Fig. 2, inset). Preparations were held in a continuous, humidified, charcoal filtered air flow (1 L) coming from a glass tube, which was positioned 5 mm from the antennal preparation. Once the antennal preparation and EAG equipment were in position the microscope light was switched off to reduce noise.

#### Electroantennography (EAG)

The test substance in solution (1 µL), was applied to a filter paper strip (4 × 60 mm) using a disposable micropipette (Microcaps, Drummond Scientific Co., USA) at 100% or 33.6 BBEXPH. After 30 s, to allow the solvent to evaporate, the filter paper strip was inserted into a disposable glass Pasteur pipette (150 mm, SLS Select Education, Nottingham, UK). When sealed this created a single use “odour cartridge”. A stimulus delivery device (Syntech Stimulus Air Controller CS-O5), which was controlled by a foot pedal, generated a “puff” of air as the tip of the odour cartridge was inserted into the air flow. For 2 s, air was passed through the odour cartridge into the main air flow, which then went over the antennal preparation. The stimulus delivery device ensured a continuous air flow by compensating for the air coming through the pipette. The application of a 1 mV pulse was performed at the start of each experiment to allow for calibration of EAG responses. Amplification of the signals was set at ×10,000. Signals were analysed using the computer-based software package, EAG 2000 (Syntech, The Netherlands^62^). Controls consisted of a positive control of ammonia, applied as 35% ammonium hydroxide (FSA Laboratory Supplies, Loughborough, UK), and its corresponding negative control, distilled water. An additional negative control of re-distilled diethyl ether was necessary for comparison with extracts. Controls were completed at the start and end of each experiment and at regular intervals throughout. The positive control (ammonia) and its negative control (water) were tested immediately, i.e. without the 30 s evaporation period.

#### EAG responses to a volatile extract from bed bug-exposed paper

EAG responses of males and females of *C. lectularius* to the volatile extract from an air entrainment of bed bug-exposed papers were recorded to determine EAG-activity of the extracts. Three males and three females were prepared as above and tested with a 1 μL of each control (ammonia, distilled water, and re-distilled diethyl ether) and the volatile extract. The antennae were stimulated once every two minutes for maximum antennal recovery and longevity (data not shown).

### Olfactometer-based behavioural assays

#### Bioassay equipment

The still-air olfactometer described by Weeks *et al*.^24^ was used for the behavioural assays. Clean cotton gloves were worn when handling olfactometers and other equipment. New pots and mesh, which were washed before use, were used for each replicate. All equipment was washed with warm water and detergent (1% Teepol 12–20 unperfumed detergent), rinsed with 50% ethanol, followed by water and dried. New unexposed filter papers were used for each replicate.

Data were recorded using the video recording equipment, set-up and software as described in Weeks *et al*.^24^. EthoVision Version 3.1 software (Noldus Information Technology, Wageningen, Netherlands) was used to capture video images to track bed bugs during behavioural assays. After setting up the equipment, an individual bed bug was introduced into the centre of the arena using a fine paint brush. The duration of the bioassay was 20 min.

#### Preparation of the odour and no-odour pots

The positive control consisted of an odour pot containing a single bed bug-exposed paper, which was prepared as described above, compared to a no-odour pot containing an unexposed filter paper (accordion folded, 70 × 40 mm, Whatman 125 mm). The negative control consisted of two no-odour pots, but the filter paper was treated with re-distilled diethyl ether (10 µL). The volatile extracts and synthetic blends were removed from the freezer one hour prior to the first bioassay and were maintained at room temperature throughout the day. The volatile extract or synthetic blend (10 µL) was applied onto the unexposed filter paper in the designated odour pot and the corresponding no-odour pot was treated with re-distilled diethyl ether (10 µL).

The position of the odour and no-odour pots was randomised between replicates. For the positive control, the equipment was set up 20 min before the start of the bioassay. When testing the response of bed bugs to solvent, volatile extracts, and synthetic blends, the pots were left for 30 s to allow the solvent to evaporate, before the olfactometer was assembled.

#### Preparation of the volatile extracts

As the volatile composition released by a bed bug-exposed paper during a behavioural assay has been found to be attractive to bed bugs previously^23,24^, the bed bug-exposed paper hours (BBEXPH), which refers to the time of air entrainment, is reported. The 100% extract, which is equivalent to 33.6 BBEXPH per uL, was diluted in re-distilled diethyl ether to test the response of bed bugs to various concentrations. The application of 10 uL was taken into consideration. In behavioural assay 1, three concentrations were tested, 0.0675 (0.02%), 0.675 (0.2%), and 6.75 (2%) BBEXPH. In behavioural assay 2, two more concentrated solutions were tested alongside the 6.75 BBEXPH, 13.50 BBEXP (4%) and 27.00 BBEXP (8%) alongside the synthetic blends. The solutions were stored in 1.1 mL pointed vials (Chromacol, Welwyn Garden City, UK) at −20 °C prior to use in bioassays.

#### Behavioural assay 1: concentration response with the volatile extract from bed bug-exposed papers

The response of bed bugs to different concentrations of the volatile extract from bed bug-exposed papers was examined by behavioural assay. Treatment (i.e. negative control (solvent), positive control (bed bug-exposed paper) and three volatile extract concentrations), sex, and odour pot position were considered as factors and randomised. In each block (comprising one day) males and females of *C. lectularius* were tested with each of the five treatments. Therefore, one block comprised 10 bioassays (n = 20). Response variables were: number of visits to pots and time spent in zones (see inset in Fig. 1). The bioassays were completed in the early scotophase, between 10:00 and 15:00.

### Identification of GC-EAG-active compounds

#### Coupled gas chromatography-electroantennography (GC-EAG)

The GC used was an Agilent-6890N, containing a HP-1 column (30 m × 0.32 mm; film thickness, 0.52 μm; J & W Scientific, Folsom, CA, USA) with a COC injector, hydrogen carrier gas and an FID. The oven temperature was maintained at 40 °C for 2 min and then programmed at 5 °C/min to 100 °C and then at 10 °C/min to 250 °C. The end of the column was inserted into the centre of the main air flow so that the eluent went over the antennal preparation. A heated transfer line ensured that the compounds did not cool and condense inside the column once they left the GC oven. Amplification of the signals was set at ×10,000. Signals were analysed using the computer-based software package, EAD 2000 (Syntech, The Netherlands^62^). The volatile extract (1 μL, at 100% or 33.6 BBEXPH) from an air entrainment of bed bug-exposed papers was injected onto the column.

Ten male preparations and ten female preparations for a total of 20 GC-EAG runs were completed. GC-EAG-active peaks from the coupled GC-EAG analysis were determined by comparing traces visually on a light box to match corresponding EAG responses to peaks. Responses were matched between males and females separately onto master traces for each sex. Where there were five or more responses in the same place on the electroantennograms, the corresponding place on the chromatogram was marked and the retention time of the peak was recorded. Master traces of males and females were then overlaid to identify where responses occurred in both sexes. A solution of n-alkanes (C_7_-C_25_, 1 μL of 100 ng/μL) in hexane also was analyzed to calculate the retention indices (RI) of any GC-EAG-active peaks for comparison with GC and coupled GC-MS traces^61^.

#### Coupled gas chromatography-mass spectrometry (GC-MS)

Volatile extracts (1 μL at 100% or 33.6 BBEXPH) from the bed bug-exposed paper and two control air entrainments were analysed by coupled GC-MS, using a Micromass Autospec Ultima magnetic sector MS, coupled to an Agilent 6980 GC equipped with a HP-1 capillary column (50 m × 0.32 mm; film thickness, 0.52 µm; J & W Scientific, Folsom, CA, USA) and fitted with a COC injector. The carrier gas was helium. The oven temperature was maintained at 30 °C for 5 min, and then programmed at 5 °C/min to 250 °C. Ionization was by electron impact at 70 eV, source temperature 220 °C. Electrophysiologically-active peaks found to be specific to volatile extracts from bed bug-exposed papers, but absent from controls, were tentatively identified by comparison with MS databases (NIST, 2002) and confirmed by co-injection for peak enhancement on the two columns with stationary phases of differing polarity^63^. Once confirmed, multiple point external standards were used to quantify each of the GC-EAG-active compounds identified from the air entrainment extract of the bed bug-exposed paper (n = 3). Most of the identified compounds were purchased commercially from a variety of sources (≥95% purity). Compounds that were not available commercially, i.e. β-phellandrene (90% purity), (*S*)-germacrene D (92% purity), and (*3E,5E*)-octadien-2-one (83% purity), were provided via liquid chromatography/chemical synthesis. (*R*)-Germacrene D (11% by GC) was purified from “gum haggar”, a gum resin from *Commiphora holtziana* (Engl., 1904)^64^, whilst (*S*)-germacrene D (92% pure) was purified from a commercially available sample (RC Treatt, UK, 40% pure) by small-scale liquid chromatography over silica impregnated with silver nitrate. The two enantiomers of germacrene D were then combined in solution in equal amounts for co-injection. 3,5-Octadien-2-one (40 mg, 83% pure by GC) was synthesized in one step starting from (*E*)-2-pentenal (Sigma-Aldrich, Dorset, UK, 95% pure)^65^. β-Phellandrene was obtained from a commercial source (no longer in production) as used by Barata *et al*.^66^.

### Behavioural testing of synthetic blends

#### Preparation of the synthetic blends

For behavioural assay 2, the concentration response experiment, a 16-component synthetic blend (SB-16) was tested that contained the identified GC-EAG-active compounds except for those that needed to be synthesized or purified before they could be quantified and used in behavioural assays (Table 1). All compounds were obtained commercially and were added to the blend in the amounts calculated by the multiple point external standard method (Table 1). Each compound was prepared in a 1 or 5 mg/mL stock solution and then added to a concentrated mixture in the correct ratios. Gas chromatography on HP-1 and DB-WAX columns (methods described earlier) was used to check that the ratios of the chemicals in the synthetic blends were accurate before they were diluted to 6.75 (2%), 13.50 (4%), and 27.00 (8%) BBEXPH. The synthetic blends were diluted in freshly re-distilled diethyl ether and stored in 1.1 mL pointed vials (Chromacol) at −20 °C prior to use in bioassays.

For behavioural assay 3, to compare different synthetic blends, an 18-component (SB-18) and a refined 6-component (SB-6) synthetic blend also were prepared (Table 1) and all three were tested at 27 BBEXPH (8%). SB-18 contained the 16 chemicals included in SB-16 and, additionally, the two chemicals that were provided via liquid chromatography/chemical synthesis, (*S*)-germacrene D and (*3E,5E*)-octadien-2-one. SB-6 contained the six compounds that gave statistically significant GC-EAG responses following the Sloane and Sullivan method for automatic detection^67^.

#### Behavioural assay 2: concentration response with SB-16

The response of bed bugs to different concentrations of SB-16 was examined by behavioural assay. The factors were treatment (i.e. negative control (solvent), positive control (bed bug-exposed paper), three concentrations of the volatile extract and the same three concentrations of SB-16 (6.75, 13.50, 27.00 BBEXPH)) and odour pot position. The order of testing and the odour pot position were randomised within each block. In each block (comprising one day) males of *C. lectularius* were tested with each of the eight treatments. Therefore, one block comprised eight bioassays (n = 20). Response variables were: number of visits to pots and time spent in zones. The bioassays were completed in the early scotophase, between 10:00 and 15:00 (see inset in Fig. 1).

#### Behavioural assay 3: testing synthetic blends

In addition to SB-16, two other synthetic blends were tested, SB-18 and SB-6. The factors were treatment (i.e. negative control (solvent), positive control (volatile extract at 8% or 27.00 BBEXPH), and the three synthetic blends, SB-18, SB-16, and SB-6, all at 8% or 27.00 BBEXPH), and odour pot position. The order of testing and the odour pot position were randomised within each block. Two blocks were completed each day, and, in each block, males of *C. lectularius* were tested with each of the five treatments. Prior to the start of behavioural testing, an external positive control was completed with the bed bug-exposed paper to ensure that any lack of attraction in the treatments was not due to external conditions. Therefore, one block comprised five bioassays and each day 11 bioassays were completed, i.e. two blocks plus the external positive control (n = 20). Response variables were: number of visits to pots and time spent in zones (see inset in Fig. 1). The bioassays were completed in the early scotophase, between 10:00 and 15:00.

### Statistical analyses

All EAG responses were standardized by dividing the absolute amplitude by the 1 mV calibration recording. For normalization, the negative controls, before and after each test stimulation, were averaged and the difference between this value and the corrected EAG response (mV) to the compound or extract was calculated. Electrophysiological activity of the air entrainment extract was determined by comparing the mean difference between the EAG response (mV) to the volatile extract and the negative control by using a paired t-test.

Chemicals were quantified by plotting the concentration (ng/µL) against the peak area and performing a logistic regression analysis on the amount of standard chemical injected and the corresponding area of the peak to give a calibration curve. Using the equation from the response curve the amount of each chemical within 1 µL (33.6 BBEXPH) of the volatile extract was then calculated.

For analysis of the behavioural assays, tracks where the bed bug failed to move any distance greater than from the centre of the arena to the edge (non-responders), or when the equipment failed and the bed bug was inaccurately tracked (failure of software), were excluded from the analysis. Non-responders were equal to less than 4% of insects. The failure rate of the EthoVision software was less than 5%. Furthermore, all tracks within a block were excluded if there was no response to the positive control of bed bug-exposed paper. In this case, no response was defined by no visits to either pot or more visits to the no-odour pot.

EthoVision was used to analyse the x, y coordinates to calculate variables following Weeks *et al*.^24^. Then the data were exported and the difference between the odour and no-odour data (O-NO) was calculated. A positive average value, therefore, indicated a greater mean for the odour than the no-odour zone or pot. The difference in the time spent in each zone (in s) and the difference in the number of visits to each pot was calculated. Least significant differences (LSDs) at the 5% level were used to compare means between treatment factors to determine significance. Treatment means were compared to the negative control (labelled ‘a’ if significantly different), bed bug-exposed paper (labelled ‘b’) and volatile extract from bed bug-exposed paper (labelled ‘c’).

The data were normally distributed as indicated by the residual plots for each response variable. However, the dataset contained several missing values due to occasional tracking issues, which resulted in an unbalanced design. Therefore, a restricted maximum likelihood (REML) method was used to estimate effects. The null hypotheses were that there was no significant difference in the behaviour of bed bugs in the presence of paper that had been previously exposed to conspecifics (bed bug-exposed paper), volatile extracts of this paper, or synthetic blends mirroring the volatile extract compared with the controls.

All statistical analyses of all data collected during this study was completed using GenStat version 11.0^68^.

## Data Availability

All data generated or analysed during this study are included in this published article or are available on request from the corresponding author.
